# Intracatheter Tissue Plasminogen Activator for Chronic Subdural Hematomas after Failed Bedside Twist Drill Craniostomy: A Retrospective Review

**DOI:** 10.7759/cureus.6472

**Published:** 2019-12-26

**Authors:** James Brazdzionis, Tye Patchana, James G Wiginton, Margaret Rose Wacker, Rosalinda Menoni, Dan E Miulli

**Affiliations:** 1 Neurosurgery, Riverside University Health System Medical Center, Moreno Valley, USA; 2 Neurosurgery, Arrowhead Regional Medical Center, Colton, USA

**Keywords:** subdural hematoma, subdural drainage, subdural, tpa, tissue plasminogen activator, chronic, chronic subdural hematoma, twist drill craniostomy, safety, efficacy

## Abstract

Introduction

Chronic subdural hematomas (cSDH) are common in neurosurgery with various symptoms and significant morbidity and mortality. Treatment varies with procedures including twist-drill (TD) craniostomy, craniotomy, burr hole craniostomy, and craniectomy. Newer treatments including middle meningeal artery embolization are also being explored as no treatment has been determined to be optimal. Due to the lack of consensus treatment, tissue plasminogen activator (tPA) has begun to be investigated to promote drainage and has shown promise in some early studies in reducing recurrence rates. We retrospectively reviewed patients who underwent TD craniostomy and received intracatheter tPA to evaluate the safety and efficacy of this practice.

Methods

A single-center retrospective review from December 2018 through August 2018 occurred for patients with cSDH 18 years of age or older who underwent a bedside TD craniostomy. Inclusion criteria included all patients who underwent treatment with TD craniostomy for drainage of cSDH during the time period in which tPA protocol was adopted as a possible therapeutic measure at our center. Exclusion criteria included all patients less than age 18 or incarcerated. Patients were stratified into two groups those that received tPA per our center's neurosurgical protocol and those that received drainage alone. Data collected included demographics, hospital/intensive care unit (ICU) length of stay, operative intervention, cSDH thickness throughout stay, length of drainage, and Glasgow Coma Scale (GCS) on arrival and discharge with analysis performed using *t*-tests.

Results

In all, 20 patients met inclusion: six received tPA at 48 hours per the institutional neurosurgical protocol and 14 did not. The average thickness of cSDH on arrival was significantly larger in the tPA group (26.5 mm vs 14.46 mm, *p *= 0.0029). Arrival and discharge GCS, average daily drainage, length of stay parameters, and percent change in thickness did not differ between tPA and no tPA groups. The average daily drainage was significantly less prior to the administration of tPA in the tPA group than in the cohort of not receiving tPA (30.71 mL vs 68.99 mL; *p *= 0.011). Average drainage in patients who received tPA after administration was significantly higher compared to pre-tPA values (131.39 mL vs 30.71 mL; *p *= 0.046). No patients were readmitted for re-accumulation or required an operating room procedure. There were no adverse outcomes identified through the instillation of tPA.

Conclusion

Intracatheter tPA increased drainage rates in the assessment of pre- and post-tPA values when administered at 48 hours after subdural drain (SDD) placement. Patients who received benefits from tPA tended to have larger subdural hematomas and less drainage prior to the instillation of tPA than patients that benefited from drainage alone. Larger prospective studies should investigate early treatment with tPA to identify if tPA is efficacious for all patients after TD craniostomy and to optimize patient selection with regard to thrombolytic therapy.

## Introduction

Chronic subdural hematomas (cSDH) are a common neurosurgical condition with an estimated incidence of 1-20 per 100,000 [1-3]. First reported in 1657 after a necroscopy, the term chronic subdural hematoma began widespread use in 1925 [4]. Patients present variably, ranging from a headache to a profound neurologic deficit [2]. This condition is associated with significant morbidity and mortality with predictive factors of age, with worse outcomes greater than the age of 60, Glasgow Coma Scale (GCS) at presentation, with patients with more optimal outcomes with a GCS greater than 8, and comorbid conditions [5]. Treatment of cSDHs varies, but since the 1970s and 1980s, it has been established that twist drill (TD) craniostomy for subdural drain (SDD) placement is a viable and efficacious option for treatment [6-8]. Additional surgical options include craniotomy as well as burr hole craniostomy [1,5]. With multiple treatment options, consensus has not yet been determined, however many surgeons prefer SDD placement as well as continued delayed drainage after burr hole craniostomy over craniotomy due to some literature noting lower rates of recurrence with continuous drainage [9]. Higher volumes of drainage have additionally been found to result in decreased rates of recurrence [10]. Thrombolytics such as tissue plasminogen activator (tPA) and urokinase have been studied as a methodology of treatment for other intracranial processes including intraventricular and intraparenchymal hemorrhage [11, 12]. Two studies have identified lower rates of recurrence and increased total drainage volume in patients who have undergone either TD or burr hole craniostomy for treatment of SDH with supplementation of tPA [10,13]. Further supporting this practice of adjunctive tPA, Frenkel et al. report a case of resolved recurrent subdural hematoma with treatment with tPA through SDD after multiple attempts of evacuation including craniotomy, TD and burr hole craniostomy [14]. The aim of this study was to add to the literature and compare the efficacy of TD alone versus TD and intracatheter tPA through measurements of volume drained throughout hospital stay and changes in thickness. Additionally, we aimed to investigate adverse events as well as differences in quality measures such as length of stay and duration of SDD that have not been previously identified. A comparison was also made between those who received tPA and those who did not to identify if any pre-dosage characteristics may be identified for appropriate patient selection and intervention with tPA.

## Materials and methods

The internal neurosurgical database of a single institution was reviewed retrospectively. Internal Review Board approval was obtained to collect data on all patients who presented with cSDHs who underwent bedside SDD placement. The need to obtain informed consent was waived by the institutional review board in light of the retrospective nature of this study. Inclusion criteria included all patients with cSDHs who were admitted to our center and underwent SDD placement. SDD placement occurred at bedside by TD craniostomy through our center's standard technique, described below. Exclusion criteria included patients who were younger than 18 years of age or incarcerated. Additional exclusion criteria included all patients treated with SDD placement prior to institutional adoption of tPA protocol to ensure appropriately matched treatment and control groups.

A surgical technique was done with local anesthetic with or without conscious sedation. Patients are typically placed supine, and the incision site is marked, usually near the parietal boss; however, an incision site may be modified based on individual characteristics and location of the subdural hematoma on pre-operative imaging where-in the incision site would be marked after obtaining measurements from preoperative scan and anatomical landmarks such as the pinna. TD craniostomy is performed at this time with a pilot hole drilled and then redirected obliquely. Dura is opened and then the subdural catheter is inserted. The subdural fluid is typically withdrawn at this time using a syringe connected to the implanted catheter and then connected to the suction bulb. Post-procedure imaging was obtained routinely.

After placement of SDD and drainage for 48 hours, repeat head computed tomography (CT) scans for these patients were evaluated by our center's attending neurosurgeons. Based on imaging characteristics and clinical examinations, these patients received tPA through the SDD through our center's neurosurgery protocol. The decision to administer tPA after the initial 48-hour drainage period was made by the attending neurosurgeon after identifying the lack of improvement of mass effect, significant residual hematoma or persistent symptoms referable to residual hematoma. The administration dosage for our protocol is 2 mg/mL given in 1-2 doses 12 hours apart. After giving tPA repeat head CT was obtained in a delayed fashion to evaluate for improvement in the subdural size. Data collected included demographic data of age, sex, and race, date of SDD placement, date of SDD removal, length of stay in the hospital, whether or not the patient received tPA through the SDD, Glasgow Coma Scale (GCS) on arrival and discharge, thickness of subdural hematoma in millimeters (mm) measured in radiology reports, inpatient records or, if size was unavailable on inpatient records, occurred through independent measurement through review of imaging. The thickness of subdural hematoma was measured on arrival, prior to placement of SDD (if different), after placement of SDD, at 48 hours after SDD placement, prior to tPA, and prior to discharge to evaluate thickness changes of the SDH with or without tPA adjunct. Other data measured was total drainage from the SDD, drainage during placement, drainage at SDD day 1, drainage prior to tPA administration, and drainage after tPA administration. Drainage data was averaged over the total drainage period and measured in cubic centimeters (CCs). Calculations were completed to analyze the percent change of the thickness of the subdural hematoma at each interval measured. Microsoft Excel® was utilized to conduct statistical analysis with *t*-tests.

## Results

A total of 20 patients were included in the study for analysis and stratified into two groups: those who received tPA through the SDD and those who did not receive tPA through the SDD. There were no significant differences identified in preadmission anti-platelet or anticoagulant usage. No patients from either group had a documented history of subdural hematoma prior to admission. Demographics data from the two groups are presented below in Table 1. 

**Table 1:** Demographic data for the study participants

	Tissue plasminogen activator (N = 6)	No tissue plasminogen activator (N = 14)	P-value
Average Age (years)	69.33	68.43	0.933
Race			
Caucasian	1	4	
Hispanic	4	7	
Asian	1	0	
African American	0	3	
Sex			
Male	4	10	
Female	2	4	
History of Trauma (Number of Patients)	4	10	0.850
Blood Thinner Usage (Number of Patients)	4	6	0.534
Average Months Post-Procedure	10.33	11.07	0.150
Number of Patients with Follow-up Neurosurgery Clinic Visits	2	3	0.632

A retrospective review of the two groups identified that the average thickness of the subdural hematoma on arrival was significantly higher in the tPA group than the group that did not receive tPA through the SDD. The average thickness on discharge, GCS on arrival, GCS on discharge, percent change of thickness of the subdural from admission to discharge, average daily drainage, SDD output on day 1, and the average length of stay did not differ between groups. Furthermore, as seen in Table 1, no patients from either group required re-admission at our center for re-accumulation of the subdural hematoma and no patients from either group were brought to the operating room for a craniotomy. Neurosurgery clinic follow-up was limited with only three patients following up as outpatients in the no tPA group and 2 patients from the tPA group. The average number of months post-procedure without identified readmission at the facility of the original procedure is presented in Table 1. This data point is limited as it does not include admissions to alternate facilities. It is important to note that three patients from the no tPA group were removed from analysis for the length of stay due to complications of acute hypoxemic respiratory failure, acute hypoxemic respiratory failure secondary to pneumonia, and a DVT and psychosocial factors complicating patient placement. Please see Table 2 for a summary of this data.

**Table 2:** A comparison of outcomes from the tissue plasminogen activator and no tissue plasminogen activator groups

	Tissue plasminogen activator	No tissue plasminogen activator	P-value
Average Thickness of Subdural Hematoma on Arrival (mm)	26.5	14.46	0.0029
Average Thickness of Subdural Hematoma on Discharge (mm)	12.02	7.06	0.052
Glasgow Coma Scale on Arrival (Average)	14.67	13.86	0.27
Glasgow Coma Scale on Discharge	14.67	13.07	0.11
Percent Change of Thickness of Subdural Hematoma on Discharge	52.04	43.04	0.51
Average Daily Drainage from Subdural Drain (mL)	66.43	68.99	0.912
Average Length of Subdural Drain (Days)	4	3.57	0.56
Subdural Drain Day 1 Output (mL)	41.33	80.71	0.19
Average Length of Stay (Days)	6.67	10.25	0.25
Readmission for Re-accumulation of Subdural Hematoma (number of patients)	0	0	
Craniotomy for Subdural Hematoma (number of procedures)	0	0	

Although average daily drainage did not differ between the two groups, the pre-tPA and post-tPA average daily drainage was significantly increased after receiving tPA. Further, the average daily drainage in the tPA group prior to receiving tPA was significantly less than that of the no tPA group. The average percent change of the cSDH thickness pre- and post-tPA did not differ (as evaluated at the 48-hour mark in the pre-tPA group using pre-procedure and 48-hour CT scans, and by the percent change from initiation of protocol to discontinuation of protocol in the post-TPA group measured by serial CT scans), nor did the average percent change of the cSDH thickness comparing pre-tPA (measured at the 48-hour mark from initial to 48-hour CT scans) to the no tPA group (measured from the initial scan to the discharge scan). The average drainage from the post-tPA group was higher than that in the no tPA group but was not statistically significantly higher. Please see Tables 3-5 for a summary of this data. 

**Table 3:** A comparison pre- and post-tissue plasminogen activator drainage values

	Pre-tissue plasminogen activator	Post-tissue plasminogen activator	P-value
Average daily drainage (mL)	30.71	131.39	0.046
Average percent change subdural hematoma thickness	33.32	20.12	0.38

**Table 4:** Comparing drainage and thickness values pre-tissue plasminogen activator to the no tissue plasminogen activator group

	Pre-tissue plasminogen activator	No tissue plasminogen activator	P-value
Average daily drainage (mL)	30.71	68.99	0.011
Average percent change subdural hematoma thickness	33.32	43.04	0.49

**Table 5:** Comparing post-tissue plasminogen activator drainage to the no tissue plasminogen activator group

	Post-tissue plasminogen activator	No tissue plasminogen activator	P-value
Average daily drainage (mL)	131.39	68.99	0.22

## Discussion

Management of cSDHs has been controversial with differing opinions on procedures offered for treatment, and with intrinsic risks of morbidity and mortality, optimizing treatment has been desired throughout the literature [7]. Though there is limited literature on the treatment of cSDHs with tPA and thrombolytics, some literature supports consideration for its utilization [10,12-13]. As this is a frequent occurrence in neurosurgical centers, effective treatment options should be investigated and utilized.

Our data identified that patients who presented with cSDHs were overall effectively treated with TD craniostomy, consistent with reports throughout the literature [6-7,9]. However, what differed is our utilization of tPA. Although one study investigated its efficacy in TD craniostomy and burr hole drainage and another reported the effectiveness of tPA after craniotomy, we believe our study adds to the literature identifying potential benefits to optimal therapy for subdural hematomas with thrombolytics [6,10].

In our studied population, patients with cSDHs who underwent drainage augmented with tPA tended to present with larger subdural hematomas than in the no tPA group, otherwise presenting characteristics including GCS did not significantly differ. Age, anti-platelet and anti-coagulant usage, and history of trauma also did not differ between groups.

**Figure 1:**
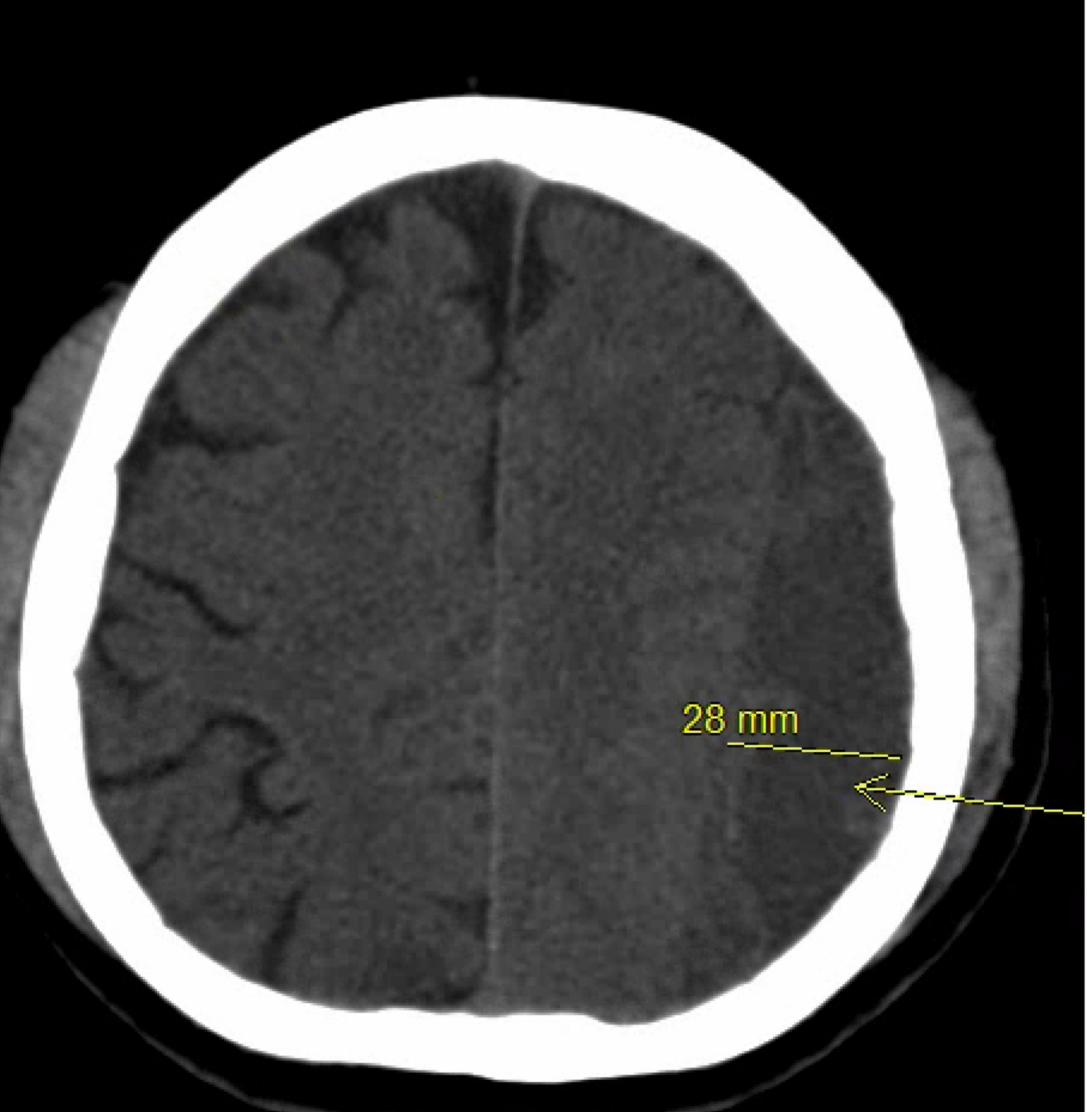
Pre-drainage CT scan of a patient who underwent subdural drain placement with tissue plasminogen activator adjunct Thickness was measured at approximately 28 mm; the arrow is pointing to the location of the subdural hematoma providing significant mass effect.

**Figure 2:**
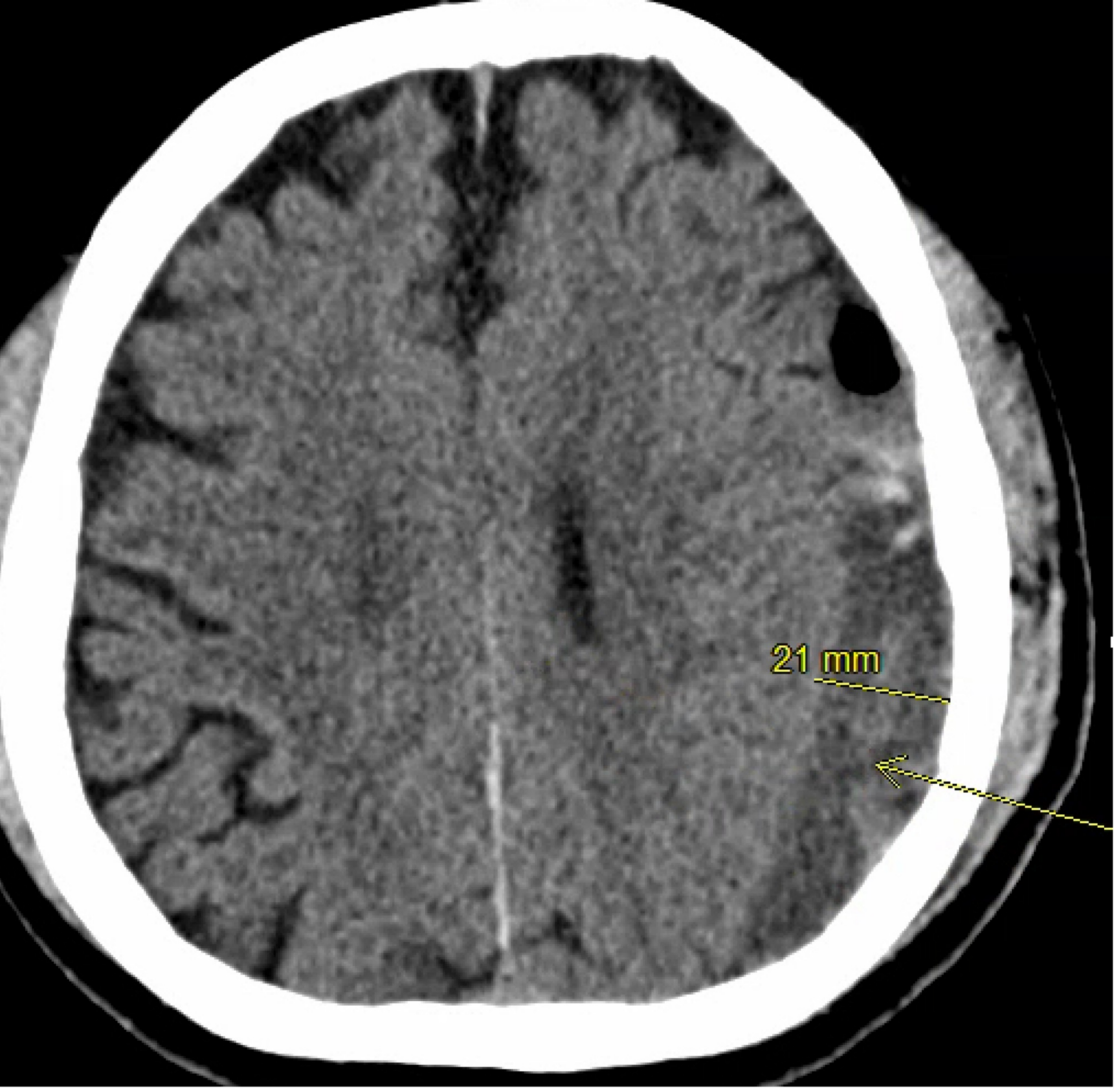
Pre-tissue plasminogen activator CT scan of a patient who underwent subdural drain placement with tissue plasminogen activator adjunct This image was taken prior to the initiating protocol for the administration of tissue plasminogen activator through the subdural drain. Mass effect is decreased but still present from the subdural hematoma, marked by the arrow compared to figure 1. Some pneumocephalus is present from initial drain placement and there is evidence of some post-traumatic hemorrhage caused by drain placement.

**Figure 3:**
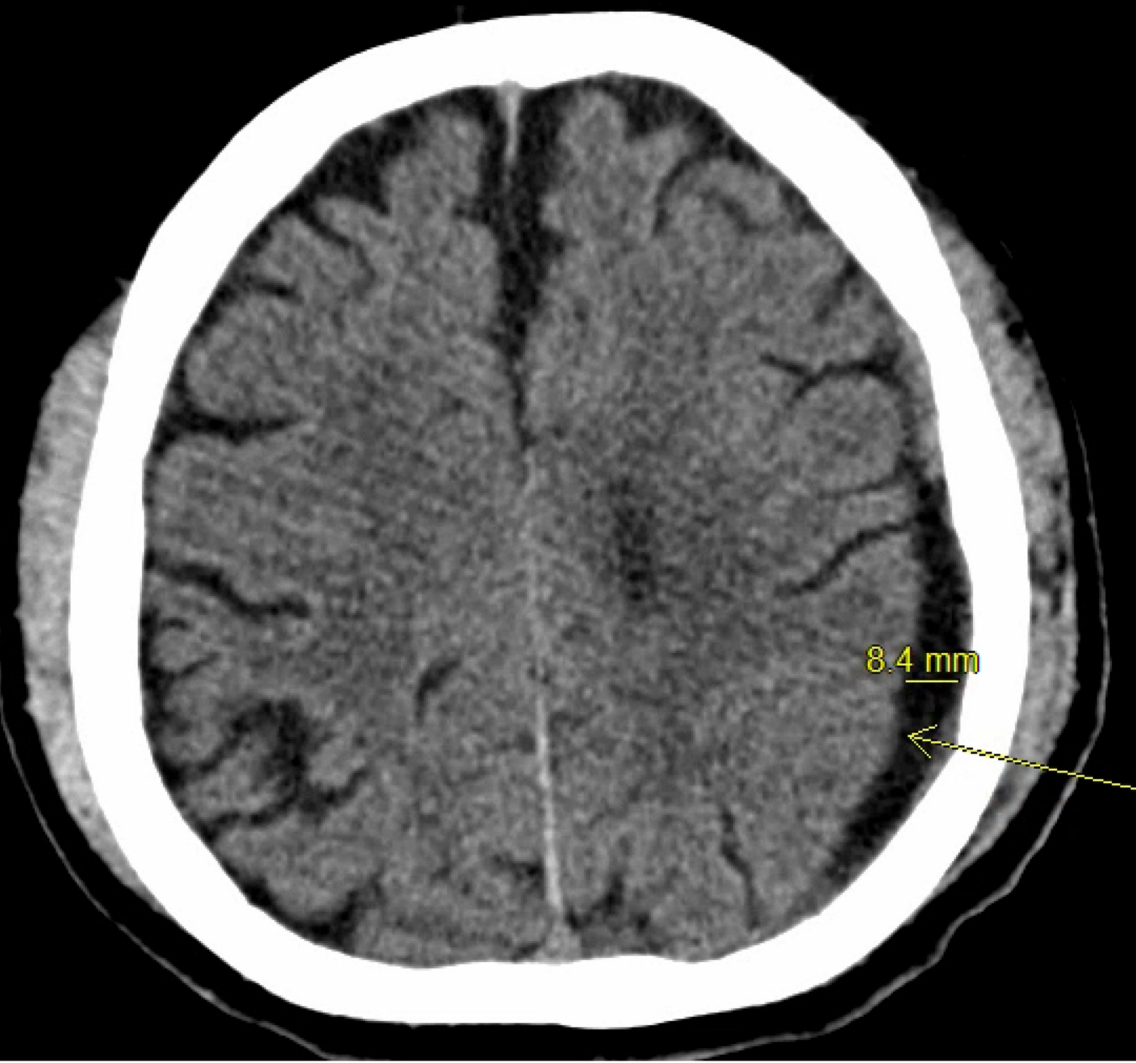
Post-tissue plasminogen activator and prior to discharge CT scan of a patient who underwent subdural drain placement with tissue plasminogen activator adjunct After the initiation of tissue plasminogen activator, the patient had increased drainage. This patient had significantly decreased mass effect and size of the subdural hematoma, marked by the arrow, after the initiation of tissue plasminogen activator protocol.

Although patients were selected for tPA per neurosurgery attending evaluation after obtaining a serial CT scan of the head, this patient characteristic may be considered as a potential factor to identify patients that may be more prone to receiving tPA. Of course, causation cannot be implied, and it cannot be assumed that all patients with large cSDHs require tPA, but it may warrant more investigation to identify if a cut-off value may be established. An example patient is found in Figures 1-3 with pre-drainage, pre-tPA and prior to drain removal CT images.

Further, this dataset identifies that there is a potential subset population that benefited from receiving tPA through the SDD. This subset group may be patients who had lower mean outputs at 48 hours who initially presented with larger subdural hematomas. This is identified through characteristics identified in our patient subset. In examining patient groups, patients who benefited from SDD placement with adjunctive tPA tended towards having larger subdural hematomas and lower mean outputs at 48 hours. This is noted through significant increases in subdural hematoma drainage prior to and post-TPA in the tPA cohort as well. Further supporting this is the relative ineffectiveness in drainage in the tPA cohort prior to administration of tPA with significantly less drainage as compared to the no tPA cohort. In patients who received tPA, the pre- and post-tPA daily average drainage outputs increased significantly. Additionally, the average daily drainage in the pre-tPA cohort was significantly smaller than that in the no tPA cohort. Overall patients who underwent adjunctive tPA in their management had non-significantly different overall drainage compared to the no tPA cohort, suggesting that within the overall drainage population, patients who do not respond to drainage may benefit from tPA adjunctive therapy to optimize their drainage. Further none of the patients in the tPA group required craniotomy or craniectomy for the evacuation of their hematoma. This suggests that patients who received tPA benefited from tPA therapy to accelerate and optimize drainage in patients who initially sub-optimally responded to drainage alone. We cannot, however, identify exact characteristics that would portend to treatment with tPA. However, it is likely that lower mean outputs at 48 hours would lead the primary surgeon to consider tPA as this study was able to show in our tPA subset that those with low outputs at 48 hours had significantly increased mean drainage after tPA.

Additional characteristics to consider in this practice is the implication of safety characteristics. No patients in either group required readmission for reaccumulation, and no patients required a procedure in the operating room after intervention. Although three patients from the no-drainage group experienced complex hospitalizations resulting in their exclusion their analysis from length of stay statistics due to conditions not related to their subdural hematoma, no patients from the tPA group experienced bleeding-related complications after instillation of the tPA. Further, the length of stay in the ICU, hospital, and length of placement of the SDD did not differ significantly between groups. This implies that treatment with tPA for cSDHs through the SDD had similar outcomes in our evaluated population than those who did not receive tPA and benefited from drain alone.

Membrane formation is also critical in understanding the pathophysiology of cSDHs. These chronic membranes have been found to be intimately related to recurrence of the subdural hematoma with postulations of recurrent neovascularization in the membrane with intermittent vessel injury leading to re-accumulation and growth [15]. Further, this re-accumulation is aided by the continuous inflammatory process from the initial injury of dural border cell damage that leads to the continuous leakage of fluid and blood from the membrane into the subdural space [16]. In patients with cSDHs, it has been identified that there are increased levels of intrinsic thrombolytics that break down the hematoma intrinsically. This intrinsic thrombolytic may be assisted by extrinsic tPA through the SDD to assist in additional hematoma breakdown to increase drainage [16]. This pathophysiological process may play a role in identifying which patients may benefit from tPA as the intrinsic thrombolytic process may affect patients with differing membrane load differently. Further, increased drainage of the liquid component of the subdural may reduce re-accumulation and therefore those with increased liquid content and less membrane may drain more effectively with drainage alone [10,16]. Further, some authors advocate for membranectomy during craniotomy to reduce rates of recurrence [17]. 

Our study is limited in that it did not investigate membrane presence on the initial imaging and whether there might be a reduction in the presence of the membrane which may be apparent on imaging, after tPA [18]. Future studies may wish to investigate these membranes and whether membranes resolved on follow-up imaging. Additional studies may also wish to investigate pre-procedural membrane load on the effectiveness of drainage and tPA. An additional limitation from this study includes our small sample size of 20 which was due to the temporal profile in which tPA was offered for cSDHs through the TD craniostomy at our institution. Other limitations of this study include limited post-procedure neurosurgical clinic follow-up. As such surrogate data were presented for the number of months post-procedure as a marker for follow-up time as none of the patients were re-admitted for recurrence of subdural hematomas at the facility of index procedure (approximately 11 months in the no tPA group and 10 months in the tPA group). Regarding reaccumulation, as patient data were collected from August to December 2018, there may not have been enough time between drainage and time of chart review for reaccumulation to occur. Data on whether these patients required re-admission at an alternative facility is unavailable. An additional limitation is that patients were selected after review by neurosurgery attending opinion for initiation on tPA protocol based on individual patient and imaging characteristics. To limit this potential selection bias, further studies with randomized controlled groups are needed. Follow-up studies should occur prospectively to continue to investigate this practice as well as to identify if tPA reduces rates of delayed craniotomy after identification of sub-optimal drainage with TD craniostomy.

## Conclusions

Outcome measures such as length of stay, GCS, and length of SDD placement between patients who received tPA and those who did not for treatment of cSDH did not differ. No patients from either group required craniotomy or additional operative management in the operating room for their chronic subdural hematoma either due to residual hematoma or expansion, nor did patients developed bleeding complications from the instillation of tPA. The patient cohort that underwent treatment with tPA had significantly increased average drain output pre- and post-tPA. Therefore, in patients who initially present with larger subdural hematomas with decreased drainage at 48 hours, the instillation of tPA may optimize drainage from TD craniostomy to the level of patients who did not require adjunct thrombolytic therapy. Further research to investigate the efficacy of early treatment with tPA in patients with cSDHs may be performed in a prospective fashion in an attempt to investigate its efficacy for all patients and identify if there are additional characteristics that can be captured to promote optimal therapy.
